# *Helicobacter pylori* and Human Immunodeficiency Virus Co-Infection: Potential Implications for Future Gastric Cancer Risk

**DOI:** 10.3390/microorganisms11040887

**Published:** 2023-03-29

**Authors:** Marcel Nkuize, Stéphane De Wit, Pieter Demetter, Pierre Eisendrath, Jean Vanderpas

**Affiliations:** 1 Saint Pierre University Hospital Brussels, Université Libre de Bruxelles, 1050 Brussels, Belgium; 2 Jules Bordet Institute, Université Libre de Bruxelles, 1050 Brussels, Belgium

**Keywords:** *Helicobacter pylori*, HIV, co-infection, gastric cancer, global estimate, prevalence, world regions, statistics

## Abstract

Objective: *Helicobacter pylori* and human immunodeficiency virus (HIV) are both pandemic infections with variable geographic prevalence rates. *H. pylori*–HIV co-infection at the regional and sub-regional levels with a perspective on gastric cancer incidence is discussed. Design: Based on PRISMA guidelines, national data for *H. pylori*, HIV, and *H. pylori*–HIV co-infection were collected for the general population through December 2019. Joint temporal and geographical data for *H. pylori* and HIV infections in 48 countries were available and used to generate *H. pylori*–HIV co-infection estimates by cross-sectional analysis. These data were compared with gastric carcinoma statistics for the same countries. Results: The estimated global prevalence rate of *H. pylori*–HIV co-infection was 1.7 per 1000 people, representing 12.6 million people. Prevalence according to region was, in decreasing order, sub-Saharan Africa 21.9‰, Eastern Europe/Central Asia 4.3‰, Latin America/Caribbean 2.0 ‰, North America/Western/Southern/Northern Europe 1.1‰, Asia/Pacific 0.8‰, and North Africa/Middle East 0.1 ‰. The incidence and mortality rates for gastric carcinoma were higher in East/Pacific Asia, Southern/Andean Latin America, and Eastern Europe regions, and the incidence appeared to be 1.8-fold greater in *H. pylori*–HIV-infected people in East Asia. Conclusions: The population at risk of *H. pylori*–HIV co-infection is estimated to be 12.6 million people (2015 reference year). The heterogeneity of *H. pylori*–HIV co-infection across regions and sub-regions does not show a clear association with gastric carcinoma. Other methodological approaches with analytical studies (cohort, case–control) are required to measure the potential effect of *H. pylori* infection and its treatment on the incidence of gastric carcinoma in the large HIV–*H. pylori*-positive cohort.

## 1. Introduction

*H. pylori* is a bacterial pathogen that is responsible for various pathologies ranging from chronic gastritis and peptic disease to gastric malignancy [1,2,3,4,5]. At the continental or subcontinental level, the prevalence of pre-neoplastic lesions and gastric carcinoma (GC) varies across populations similar to that of *H. pylori* infection, as shown in Europe and China [6,7]. An exception to this is the African paradox which is characterized by a high prevalence of *H. pylori* infection and a low GC frequency [8]. When analyzed globally, more than half of the world’s population (59.2%) is infected with *H. pylori* [9]. HIV, on the other hand, is also a global infection, with estimates indicating that in 2022, there were 38 million people living with HIV (PLHIV) [10].

While *H. pylori* infection is transmitted equally between males and females through dietary contamination during childhood, HIV is mainly acquired in adulthood through heterosexual intercourse. In both cases, *H. pylori* prevalence and HIV prevalence vary widely according to geographical region [10,11,12]. Therefore, questions have emerged regarding whether HIV infection in *H. pylori*-colonized people leads to an increased risk of gastric carcinoma.

There is a paucity of descriptive data on the association between these two endemic infections [13,14,15,16,17]. Our aim was to determine the proportion of people exposed to both pathogens according to their geographical distribution with the goal of raising awareness of the potential risk of increased GC incidence related to the possible interaction between these pathogens over the long term in the human population.

## 2. Methods

### 2.1. Search Strategy

According to PRISMA guidelines [18], electronic databases from PubMed, PubMed Central, Google Scholar, clinicaltrials.gov, ISRCTN registry, and Prospero were used to establish a database of data regarding the prevalence of *H. pylori*–HIV co-infection. Medical Subject Headings (MeSH) keywords, “human immunodeficiency syndrome” or “AIDS” AND “Helicobacter *pylori* co-infection” AND “global” AND “epidemiology” AND “database” AND “general population” were employed for a computer retrieval of published material in the English language from 1990 to 2019. Surveys measuring *H. pylori*–HIV co-infection were scarce. To circumvent this issue, since *H. pylori* and HIV are independently acquired infections, the second approach was to search for overall estimates for HIV and *H. pylori* at the country, regional, and global levels [9,10]. The MeSH keywords “*Helicobacter pylori* or *H. pylori*, national prevalence, serology, world” for *H. pylori* infections and “HIV/AIDS prevalence, country” for HIV infections were employed. In addition, hand searching and reference checking were used to increase the retrieval of missing data [19,20,21,22]. Epidemiological data on the frequencies of both pathogens were combined to reflect their geographical and temporal associations in a cross-sectional analysis.

### 2.2. Study Selection and Eligibility Criteria

**Figure 1:**
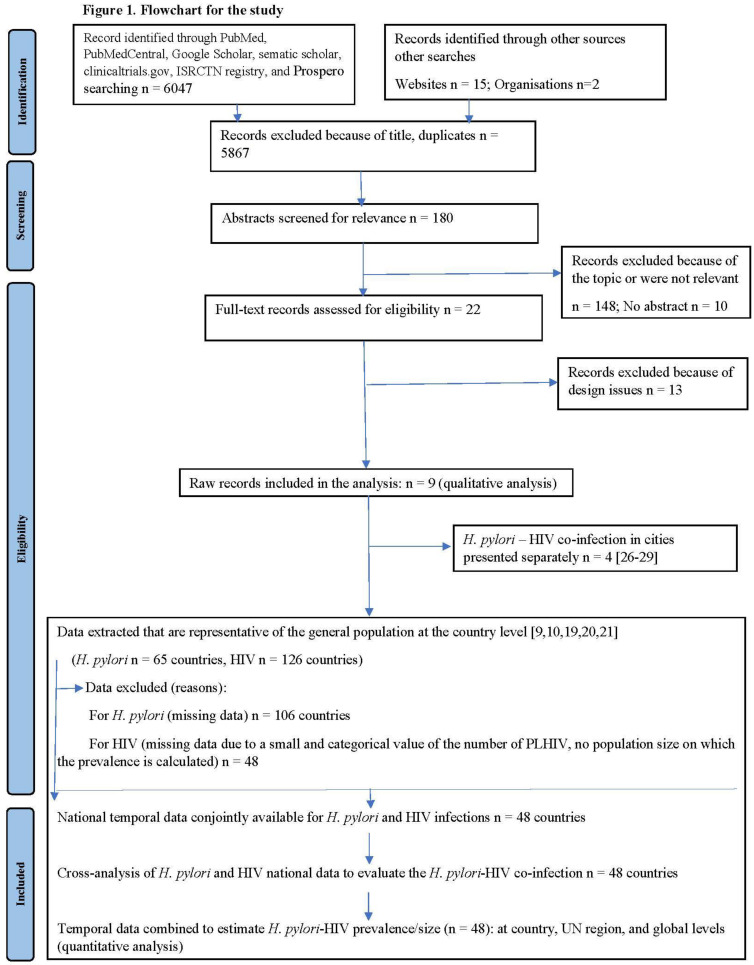
The flowchart of this study [9,10,19,20,21,23,24,25,26].

We checked all identified sources on *H. pylori*–HIV co-infection for eligibility criteria to include in our analysis, as presented in the PRISMA flowchart (Figure 1).

Inclusion criteria to select reports for *H. pylori*–HIV co-infection data were (1) representative of the general population, (2) national or international meta-analysis, and (3) serology-based diagnosis. Data that matched the following criteria were excluded: (1) endoscopy-based data from symptomatic individuals, (2) case reports, (3) experimental and case–control studies, and (4) correspondence and letters.

Inclusion criteria and exclusion criteria to select reports for *H. pylori* prevalence are presented below in the section on “*H. pylori* prevalence”.

Inclusion criteria to select reports for HIV prevalence were (1) representative of the general population, (2) national, subregional, or worldwide data, and (3) serology-based diagnosis. Exclusion criteria included reports that were single-center, not representative of the general population, and experimental studies.

### 2.3. Main Outcomes

Outcomes of interest were reports and databases representative of the general population of the country, providing temporal and geographical prevalence of *H. pylori*–HIV co-infection, *H. pylori* infection, and HIV infection, as well as the incidence and mortality of GC in the last year of the study period.

### 2.4. Data Extraction

M.N. and J.V. screened the titles and abstracts of studies identified to meet the inclusion criteria. The full texts of selected records were assessed by M.N. and J.V. for eligibility. P.D. performed an additional search. Any disagreements were resolved by consensus between M.N. and J.V.

### 2.5. Determination of H. pylori Prevalence

The method of determination of *H. pylori* prevalence was described previously by Hooi et al. [9]. Briefly, *H. pylori* prevalence for each country was estimated by pooling the data from eligible reviews. A random-effects model was used to calculate pooled prevalence estimates with 95% confidence intervals (CIs).

Concerning heterogeneity, this was assessed by stratifying the geographical variations as follows: (1) geographic region based on the United Nations (UN) classification; (2) time periods for evaluating the prevalence of *H. pylori* split into 1970 to 1999 and 2000 to 2016; (3) restriction of the analysis to adults only (aged 18 years and older); and (4) primary modality of testing *H. pylori*, including serology, urea breath test, stool antigen test, rapid urease test, or histopathology. Serology was the main diagnostic method in more than 75% (173/226) of studies.

Concerning the partitioning of the UN geographic regions, these were defined as follows: North America; Latin America and the Caribbean; Europe (Northern, Southern, Western, Eastern); Africa; Asia (Central, Eastern, Southern, South-Eastern, Western); and Oceania. Attention was paid to the indigenous populations in the United States and Australia by analyzing these separately from the general population of the country.

Concerning the prevalence over a multi-year period, this study was conducted for the period that captured the most updated data, while for multiple studies for the same country and period, the pooled statistic was calculated.

Concerning the number of people affected with *H. pylori* infection, this was extrapolated from the prevalence estimates to the total population in 2015 living in countries and regions as per the UN Population Division. It was assumed that countries with missing data in a region had a comparable prevalence to our pooled mean prevalence.

The method of *H. pylori* infection diagnosis was based on the serological determination of immunoglobulin IgG against *H. pylori*, stool *H. pylori* antigen test, urea breath test, and biopsies for rapid urease test, histology, or culture [27].

Concerning participants in studies: Individuals included had to be representative of the general population. Data from multicenter and multinational studies were analyzed separately by country and region.

Concerning the classifications of studies, there were two types: national, when the report or multicenter study involved multiple regions in the country; and sub-national, when there was only a region involved, and city level.

Clarifications with the corresponding author were made when necessary (e.g., data errors).

### 2.6. Exclusion Criteria

Exclusion criteria were of three kinds. First, according to the publication type such as guidelines, perspectives, correspondence, letters, conference abstracts, or presentations without formal publication, systematic reviews or meta-analyses, surveillance registration or nationally notifiable disease reports of *H. pylori*, and studies without defined study periods; economic analyses, modeling, time series, or transmission studies; mortality or survival analyses; diagnostic assay or test performance studies; animal studies. Second, according to the study population, such as those typically associated with a higher prevalence of *H. pylori* (e.g., patients with GC), high-risk population groups (migrants, refugees, prisoners, individuals (groups) classified as low socioeconomic status), and study participants that were restricted to selected age groups (e.g., children, elderly). Finally, according to methods of *H. pylori* diagnosis other than the four conventional tests presented above and studies not reporting the numerator and denominator on which the prevalence estimate was based.

### 2.7. Determination of HIV Prevalence

HIV prevalence represents the percentage of those living with HIV among the general population [10]. The method of determination of HIV prevalence was described previously in the UNAIDS model [28]. Briefly, for countries where HIV transmission is epidemic in the general population, available epidemiological data are drawn from pregnant women attending antenatal clinics and from nationally representative population-based surveys. Historically, in many countries, HIV sentinel surveillance was established by collecting data from a selection of clinics on a regular basis for months or years. Now, many countries have moved to using the data from routine HIV tests performed on pregnant women attending antenatal clinics. This method allows for collecting huge data sets across the country instead of single clinic data. The resulting data allow extrapolation of the national prevalence trend. While population survey data are more accurate for estimating the national HIV prevalence levels by integrating men and broader geographic coverage, they are infrequently performed. In sub-Saharan African countries where population-based surveys are not available, HIV prevalence is estimated by adjusting antenatal clinic surveillance and population-based survey data from neighboring countries.

In the other countries where HIV infection occurs mainly among key populations at higher risk of HIV and the epidemic is considered to be low-level, the estimates were drawn from surveillance among key populations, the general, low-risk population, and from HIV case reporting data, whichever were the most reliable in a given country. In countries with high-quality HIV surveillance data among the key populations, these data were used to predict national estimates and trends. Estimates of the size of key populations are increasingly derived empirically in each country; when studies were not available, they were derived based on regional values and consensus among experts. Other data sources used to estimate the HIV prevalence in the general, low-risk population included HIV case reporting data, population-based surveys, and surveillance among pregnant women.

In western and central Europe and North America, and many countries in Latin America, the Caribbean, the Middle East, and North Africa, despite insufficient HIV surveillance or survey data, they have robust disease reporting systems, HIV case reporting, and AIDS-related mortality data from vital registration systems which were directly used to provide HIV prevalence and incidence levels. The total population for each year was sourced from the 2019 Revision of World Population Prospects. Concerning categorical or missing data, correspondence with the UNAIDS was performed for clarification.

### 2.8. Gastric Cancer Incidence and Mortality

To obtain data on GC incidence and mortality, we extracted only those related to the country and region studied [29].

### 2.9. Statistics

In this study, the year 2015 was chosen as a reference because it corresponded to the most recent estimate of global, regional, and country prevalence/size of *H. pylori* infection that allowed matching with HIV data [9]. Furthermore, this *H. pylori* infection estimate was based on *H. pylori* antibodies persisting for years after the infection was cured, allowing the acceptable use of this data in epidemiological studies. Epidemiological data on the frequencies of both pathogens, *H. pylori* and HIV, were combined to reflect their geographical (country, UN region) and temporal (year) associations and then were assessed by cross-sectional analysis.

### 2.10. Data by Country for H. pylori and HIV Infections

*H. pylori* infection data extracted for analysis were estimated numbers (mean) and prevalence, population size by country, and UN region in 2015. The database from Hooi et al. estimated the size and prevalence of *H. pylori* infection by country. The estimated number of people infected with *H. pylori* was obtained by extrapolating the prevalence estimates to the total population living in countries and UN regions in 2015.

HIV data that were extracted for analysis were estimated means and prevalence by country and by UN region for adults and children living with HIV in 2015 with 95% confidence intervals (95% CI) determined by uncertainty [10,28]. The estimate of the prevalence of PLHIV was determined by dividing the number of HIV cases by the country’s population number. The method of estimating the number of people living with HIV is described in detail in UNAIDS 2019 [28].

In addition, we chose to perform separate web-based searches for HIV data for Belgium and countries with large populations, including India, Indonesia, and the Russian Federation. Data for China, a country with a large population, were included despite the wide variation per 100,000 new cases of HIV infection from one year to another [19,20,21].

### 2.11. H. pylori–HIV Co-Infection Data

We assumed that among people living with HIV, the prevalence of *H. pylori* is equal to the general population, thus:

1. Country *H. pylori*–HIV co-infection prevalence was estimated by multiplying country HIV prevalence by country *H. pylori* prevalence.
2. Country *H. pylori*–HIV co-infection size was estimated by multiplying the country (*H. pylori* size by HIV infection size) divided by the country population size.
3. Co-infection data by region or on a global scale.

Size: estimated regional or world population, HIV, *H. pylori*, and HIV–*H. pylori* co-infection population sizes were determined by summing the countries’ HIV, *H. pylori*, and HIV–*H. pylori* co-infection population sizes.

Prevalence: estimated regional population prevalence was obtained by dividing the estimated regional HIV, *H. pylori*, and HIV–*H. pylori* co-infection population sizes by the estimated regional population size.

Estimated world population prevalence was obtained by dividing the estimated world HIV, *H. pylori*, and HIV–*H. pylori* co-infection population sizes by the estimated world population size.

These data, aggregated according to the country for the year 2015, were combined in an Excel file and were matched for geographic location [9,10]. Countries with missing data for *H. pylori* (n = 106) and HIV infection data expressed as categorical without the population size used for the estimated frequency (n = 48) were excluded.

HIV infection prevalence and *H. pylori*–HIV co-infection prevalence are expressed per thousand (‰). *H. pylori* infection prevalence is expressed as a percentage (%).

The proportions by country and region underwent the Freeman–Tukey double arcsine transformation to normalize and stabilize the variance of the sampling distribution of proportions. The meta-analysis was performed on these transformed values. The values of the estimate of the true proportion and the associated confidence interval were retransformed using the harmonic mean of the sample sizes of the countries or regions.

Gastric cancer: temporal and geographic data on GC incidence and mortality in each country and region included were extracted [28].

## 3. Results

**Figure 2:**
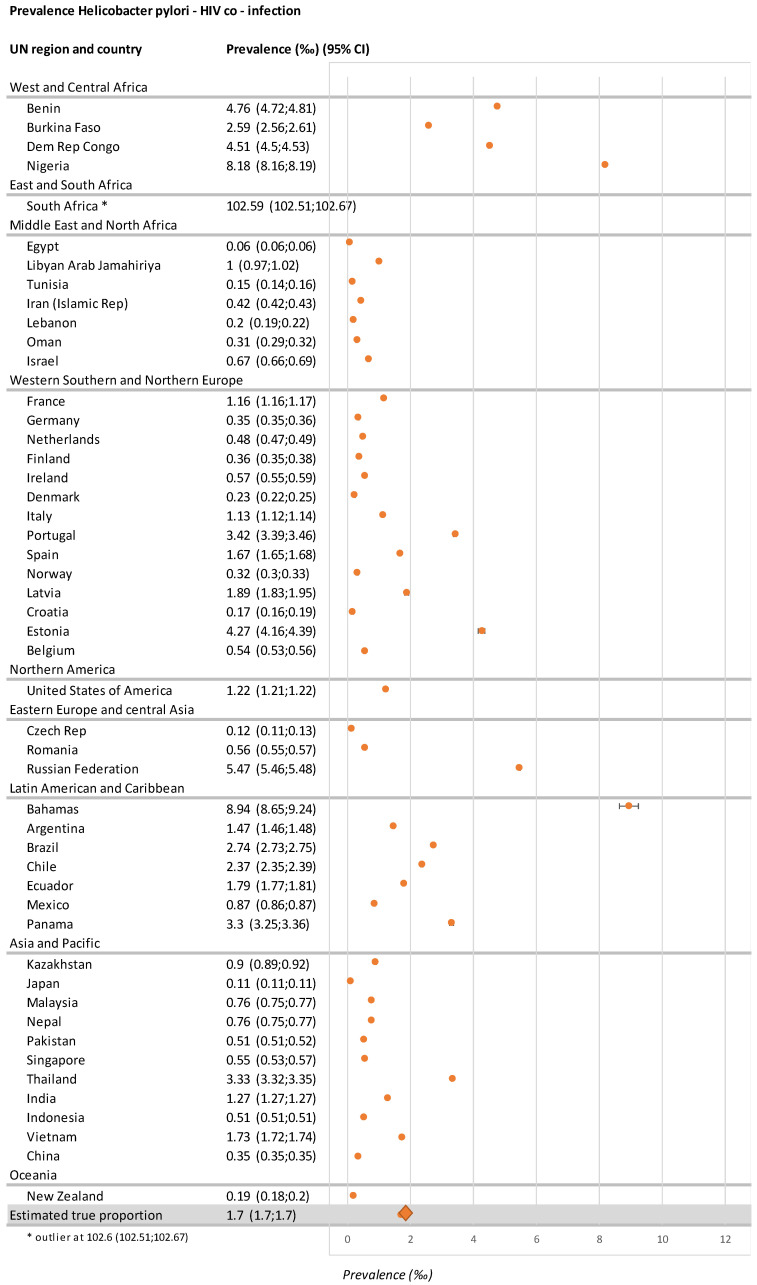
Forest plot: distribution of *H. pylori*–HIV co-infection prevalence. Each circle represents the prevalence with its confidence interval. Diamond represents the overall estimate prevalence. * Valeur very far from the others.

Of 6064 records, 180 were selected, of which nine were retained for data extraction (Figure 1). From the data in these nine records, HIV infection (n = 126 out of 171 countries) and *H. pylori* infection (n = 65 out of 171 countries) were geographically and temporally available in common for 48/171 countries (28.0%) and were used to generate estimates of *H. pylori*–HIV co-infection in 48 countries, including the UN region and global levels. The estimated global *H. pylori*–HIV co-infection rate is 1.7‰ (1.7; 1.7) based on available data from 48 countries and weighted according to the population of each country (Table 1, Figure 2).

South Africa and Nigeria are the countries with the highest prevalence of *H. pylori*–HIV co-infection. Due to the large size of the data set for each country, which is illustrated by narrow confidence intervals, there is great geographical heterogeneity. Adjustment by the weight of the inverse variance did not provide additional precision to the estimation of the true proportion.

The world human population reached 7.4 billion people in the 2015 reference year. The 1.7‰ *H. pylori*–HIV co-infection prevalence proportion corresponds to a prevalence number of 12,580,000. In addition to the data in the tables, some local reports of data deserve citation. The prevalence of the *H. pylori*–HIV co-infection in single-center studies was 32.6% in Busan (Republic of Korea in the year 2019) using the serology method, 69.7% in Tehran (Iran in the year 2011) using the stool genotyping method, and 78.1% vs. 75.1% at Assela (Ethiopia in the year 2017) using the stool antigen method among asymptomatic individuals with and without HIV infection, respectively [23,24,25]. At Bologna in Italy, the prevalence was 55% and 58% by serology among asymptomatic past-intravenous drug users versus the general population, respectively, tested by serology, and 40% and 66% among symptomatic past-intravenous drug users versus the general population, respectively, tested by pathology [26].

**Figure 3:**
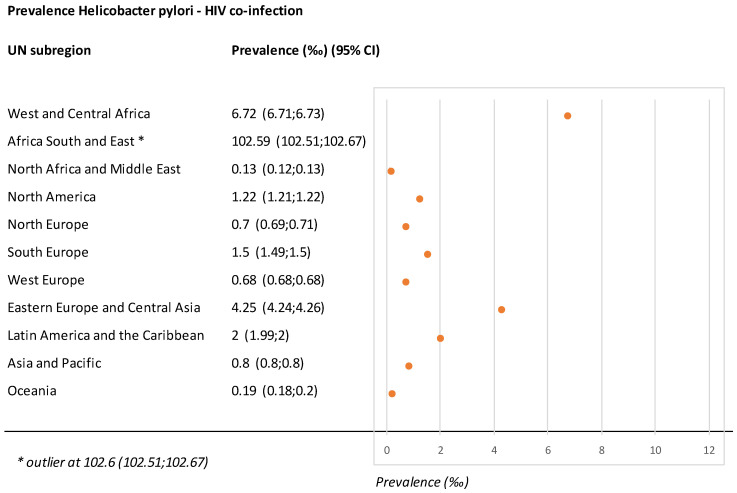
Forest plot of the *H. pylori*–HIV co-infection prevalence at the subregion level. Each circle represents the prevalence with its confidence interval. * Valeur very far from the others.

At the regional level (Figure 3), sub-Saharan Africa was the leading UN region for *H. pylori*–HIV co-infection with an estimated prevalence of 21.9‰ (21.87–21.93). The South and East Africa UN sub-regions were more affected by *H. pylori*–HIV co-infection than others.

Eastern Europe and Central Asia comprised the second-highest UN regions for *H. pylori*–HIV co-infection frequency, while the Latin America and Caribbean regions were third; the Caribbean, in particular, was the sub-region with the greatest prevalence outside Africa. North America and the Western/Southern/Northern Europe UN regions were the fourth highest for *H. pylori*–HIV co-infection, with similar prevalence rates in North America compared to Western/Southern/Northern Europe. In Western/Southern/Northern Europe, there is a North–South gradient for *H. pylori*–HIV co-infection prevalence, with Northern Europe inferior to Southern Europe. Finally, the Asia and Pacific region is one where *H. pylori*–HIV co-infection is found at a low rate. However, the current *H. pylori*–HIV co-infection prevalence in this last region is underestimated due to uncertain data from China. On the other side of the spectrum, Oceania, North Africa, and the Middle East are regions with a lower prevalence of *H. pylori*–HIV co-infection. More details are presented in Supplementary Table S1.

In terms of the number of people with *H. pylori*–HIV co-infection, the top three regions are sub-Saharan Africa, followed by Asia and Latin America, and the Caribbean (Supplementary Figure S1).

**Figure 4:**
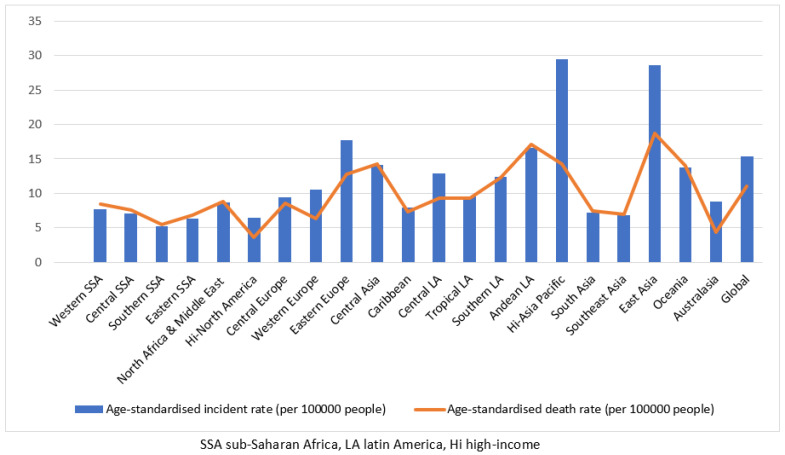
Age-standardized incidence and death rates from gastric cancer from 1990 to 2017 for 21 Global Burden of Disease regions (adapted from [29])**.** Blue columns illustrate the age-standardized incidence of gastric adenocarcinoma (per 100,000 people) across world regions, and the red curve illustrates age-standardized mortality (per 100,000 people) across world regions. The horizontal bar of 10/100,000 people age-standardized incidence. Age standardization allows comparison of populations when their age structure differs. The period studied is from 1990 to 2017.

GC incidence and death are conjointly increased in the general population in high-risk regions. Looking at the horizontal bar of 10/100,000 people age-standardized incidence (Figure 4), 10 of 21 geographical areas are characterized by an incidence of GC greater than this limit. See Supplementary Tables S2 and S3 for details. It should be noted that, from these data, no clear association appears with the distribution of *H. pylori* prevalence: for example, the two areas with the greatest GC incidence in Figure 2 (HI-Asia Pacific; East Asia and Eastern Europe) had a low 0.8% prevalence of *H. pylori*–HIV co-infection (as seen in Figure 3). Reciprocally, South Africa, with the greatest prevalence of *H. pylori*–HIV co-infection, had a low GC incidence of 5/100,000 people, as shown in Figure 4. At the present time, there is not yet clear evidence of an effect of *H. pylori*–HIV co-infection on gastric carcinoma incidence at the population level (see Section 4).

## 4. Discussion

This study assessed the prevalence of *H. pylori*–HIV co-infection at the global, regional, and subregional levels. The main findings were an estimated global prevalence of 1.7 per 1000 people and significant heterogeneity across UN regions. This heterogeneity of the distribution of *H. pylori*–HIV co-infection frequency has already been observed among the subgroup of PLHIV with symptoms justifying investigations such as an upper gastrointestinal endoscopy with gastric biopsy, serology, and other tests to diagnose *H. pylori* infection [13,14,15,16,17]. Nevertheless, this subgroup of symptomatic *H. pylori*-infected people undergoing investigation does not reflect all of the *H. pylori* carriers, given that 52% to 75% of them are asymptomatic and unaware of their infection [30,31,32].

Our estimate of the global prevalence of *H. pylori*–HIV co-infection and its heterogeneous distribution probably results from the interplay of factors such as the infection dynamics and the method of diagnosis. First, infection dynamics differ according to age, especially during childhood for *H. pylori*, notably in low-income countries (where the rate of infection is increased by low levels of education, poor access to clean water, elevated urbanization, and low socioeconomic status), and mainly during young adulthood for HIV in most countries [1,2,3,4,5]. Thus, susceptible individuals have already been infected with *H. pylori* at the time of HIV infection, while simultaneous co-infection by *H. pylori* and HIV is rare.

Second, antibiotics and proton pump inhibitors are frequently prescribed for PLHIV, and their use before tests, such as pathology, microbiology, and the urea breath test, has undermined the detection of *H. pylori* [13,14,15,16,17,23,33,34]. Moreover, the level of immune function of PLHIV appears to inversely affect the rate of *H. pylori* diagnosis by the above methods [35]. Neither medications nor normal immune function affects the performance of serology-based diagnoses (IgG titer), but antibodies against *H. pylori* disappear in the blood several years after the cure of the *H. pylori* infection and in severe immunodepression states, increasing the risk of false negative test results. Therefore, *H. pylori* serology testing appears most suitable for the epidemiological evaluation of the prevalence of *H. pylori* infection in a large population of PLHIV with caution in those with severe immunodepression [10,12,33,35,36,37].

The *H. pylori* prevalence rates discussed here are representative of the general populations of the countries or regions studied and were widely diagnosed by serology tests [9]. Moreover, HIV infection prevalence rates were based on a true evaluation of the general population (i.e., pregnant women attending antenatal clinics, population-based surveys) when HIV infection was endemic, key populations (i.e., intravenous drug users), or reported HIV cases when the prevalence in the country was low, as performed for the UNAIDS database, one of our data sources [10,28]. This accuracy of the prevalence of HIV infection, which is less frequent and a limiting factor for both microorganisms, allowed us to provide an estimated prevalence of *H. pylori*–HIV co-infection with a narrow confidence interval.

From a global view of the world’s population, the prevalence of *H. pylori*–HIV co-infection in a large population could represent a huge absolute number of *H. pylori*–HIV co-infections of epidemiological interest, notably for preventive interventions and risk projection of complications such as GC.

The interaction of *H. pylori*–HIV co-infection with gastric carcinoma risk at a population level is not yet established. It should be noted that the available data up to this point have only been descriptive. This limits the available methods of analysis, and meta-analysis methods are not appropriate for this class of descriptive data [38]. The absence of evidence of increased risk of GC at the population level in geographical areas affected by elevated *H. pylori* and HIV prevalence (as observed in South Africa) does not rule out that, in the long-term, in HIV patients, *H. pylori* could be an intermediate pathogen increasing the risk of GC [28]. Appropriate studies on the incidence of gastric carcinoma in cohorts with different *H. pylori*–HIV status are justified due to the global burden of these infections.

Some observations at the local level support the hypothesis that there is an *H. pylori*–HIV interaction with GC risk. In a tertiary hospital in Korea, a retrospective study performed between January 2004 and December 2018 detected three cases of GC in 81 PLHIV who underwent 118 screening upper gastrointestinal endoscopies and one case of GC in 192 PLHIV who underwent 192 diagnostic upper gastrointestinal endoscopies [39]. The authors estimated the incidence of GC to be 4 per 1000 patients/year for upper gastrointestinal endoscopy screening. Another study from Japan suggested at a 10-year follow-up that there was a 1.8-fold risk of GC among PLHIV compared to the general population [40]. This may be due to the factors mentioned above as well as gut dysbiosis, vitamin deficiencies, and oxidative stress, the latter being attributed to *H. pylori* reactions.

Consequently, in those regions with a high risk of GC, surveillance of preneoplastic lesions could be considered among PLHIV, given that the combination antiretroviral therapy (cART) era has led to both significant improvement in life span and increased prevalence of *H. pylori* infection [13,15,16,41]. Moreover, the global risk of cancer among PLHIV has increased, with more advanced-stage cancers and higher mortality rates. Furthermore, globally, only 15% of all GCs are diagnosed at an early stage, and GC and AIDS each caused nearly 700,000 deaths in 2018. Of those deaths, the proportion of death by GC in PLHIV is still not well known [29,42,43,44,45].

Based on this body of evidence, we suggest, first, that PLHIV with *H. pylori* co-infection should be identified and treated in adulthood but, more importantly, at a younger age, before the development of preneoplastic lesions, as *H. pylori* eradication before the advent of premalignant lesions has been shown to reduce the risk of GC [33,45]. Second, GC should be integrated as a risk factor to be screened for in those with *H. pylori*-related premalignant lesions. No recommendations exist for GC screening in PLHIV [45,46,47,48,49].

Our study has limitations and strengths. We conducted this cross-analysis of international data on *H. pylori* and HIV infections because of the paucity of national data on co-infection. Indeed, endoscopy-based data pertain mostly to symptomatic individuals and therefore exclude most *H. pylori*-infected individuals, who are often asymptomatic [30,31,32]. Our analysis did not involve “uncertainty analyses”, which concern dynamic population changes, but we performed a temporal and geographical analysis of data [9,10]. Moreover, our HIV statistics were based on a true evaluation of the general population [28]. Data for *H. pylori* infection and HIV infection were missing for several countries, and for others, they were limited. A major strength of this study is that it is the first attempt to use large databases to estimate global *H. pylori*–HIV co-infection rates, which appear to be a severe global public health burden with differences in geographical distribution. Together with this result, preliminary data from Asia suggest the need to carefully consider the risk of GC among PLHIV, particularly in regions at high risk of GC [14]. Consequently, more studies are required to document the potential for emerging excess morbidity of *H. pylori* in PLHIV, particularly in people with GC.

In summary, according to the data available, *H. pylori*–HIV co-infection is a global health burden that is estimated at 1.7 per thousand people worldwide with a heterogeneous distribution across different world regions. When analyzing the relationship at the population level of HIV prevalence, *H. pylori* prevalence, and gastric carcinoma incidence, there is, at present, no clear association between co-infection with these pathogens and gastric carcinoma. Nevertheless, the question becomes relevant to take into account the possible long-term effects of *H. pylori* in HIV-positive patients as a combined risk of GC development. Due to the more than 30 years of incubation of gastric carcinoma in *H. pylori*-positive patients, it is not surprising that an increased risk of GC in HIV-positive patients has not been documented yet [50]. There is a need for long-term cohort or case–control investigations to evaluate the risk of GC in HIV-positive patients versus HIV-negative patients. According to the precautionary principle, HIV patients should be screened for *H. pylori* infection and treated systematically and adequately.

## Figures and Tables

**Table 1:** The global estimated number of people living with HIV, *H. pylori*, and *H. pylori*–HIV co-infection based on available data for both infections from 48 countries in 2015.

	Population	HIV		*Helicobacter pylori*		*Estimated Helicobacter pylori* HIV Co-Infection		
UN region and country	Size	Size	Prevalence ‰	Size	Prevalence %	Size	Preva lence ‰	95% confidence interval lower and upper limits
**West and Central Africa**								
Benin	10,880,000	70,000	6.4	8,056,640	74.05	51,835	4.8	4.72; 4.81
Burkina Faso	18,106,000	100,000	5.5	8,475,419	46.81	46,810	2.6	2.56; 2.61
Dem Rep Congo	77,267,000	450,000	5.8	59,835,565	77.44	348,480	4.5	4.51; 4.53
Nigeria	182,202,000	1,700,000	9.3	159,700,053	87.65	1,490,050	8.2	8.16; 8.19
**East and South Africa**								
South Africa	54,490,000	7,200,000	132.1	42,306,036	77.64	5,590,080	102.6	102.51; 102.67
**Middle East and North Africa**								
Egypt	91,508,000	14,000	0.2	37,435,923	40.91	5727	0.1	0.06; 0.06
Libyan Arab Jamahiriya	6,278,000	8200	1.3	4,795,764	76.39	6264	1.0	0.97; 1.02
Tunisia	11,254,000	2300	0.2	8,191,787	72.79	1674	0.2	0.14; 0.16
Iran (Islamic Rep)	79,109,000	57,000	0.7	46,658,488	58.98	33,619	0.4	0.42; 0.43
Lebanon	5,851,000	2300	0.4	3,039,595	51.95	1195	0.2	0.19; 0.22
Oman	4,491,000	2800	0.6	2,205,081	49.10	1375	0.3	0.29; 0.32
Israel	8,064,000	7900	0.1	5,555,290	68.89	5442	0.7	0.66; 0.69
**Western, Southern, and Northern Europe**								
France	64,395,000	160,000	2.5	30,188,376	46.88	75,008	1.2	1.16; 1.17
Germany	80,689,000	81,000	1.0	28,483,217	35.30	28,593	0.4	0.35; 0.36
Netherlands	16,925,000	23,000	1.4	6,011,760	35.52	8170	0.5	0.47; 0.49
Finland	5,503,000	3500	0.6	3,124,603	56.78	1987	0.4	0.35; 0.38
Ireland	4,688,000	6200	1.3	2,015,840	43.00	2666	0.6	0.55; 0.59
Denmark	5,669,000	6000	1.1	1,254,550	22.13	1328	0.2	0.22; 0.25
Italy	59,798,000	120,000	2.0	33,606,476	56.20	67,440	1.1	1.12; 1.14
Portugal	10,350,000	41,000	4.0	8,942,400	86.4	35,424	3.4	3.39; 3.46
Spain	46,122,000	140,000	3.0	25,307,141	54.87	76,818	1.7	1.65; 1.68
Norway	5,211,000	5400	1.0	1,597,772	30.66	1656	0.3	0.3; 0.33
Latvia	1,971,000	4700	2.4	1,561,229	79.21	3723	1.9	1.83; 1.95
Croatia	4,240,000	1400	0.3	2,234,056	52.69	738	0.2	0.16; 0.19
Estonia	1,313,000	6800	5.2	1,083,356	82.51	5611	4.3	4.16; 4.39
Belgium [19]	11,299,000	18,758	1.7	3,694,773	32.70	6134	0.5	0.53; 0.56
**Northern America**								
United States of America	321,774,000	1,100,000	3.4	114,552,012	35.60	391,602	1.2	1.21; 1.22
**Eastern Europe and Central Asia**								
Czech Rep	10,543,000	3100	0.3	4,342,662	41.19	1277	0.1	0.11; 0.13
Romania	19,511,000	16,000	0.8	13,372,839	68.54	10,966	0.6	0.55; 0.57
Russian Federation [20]	143,457,000	1,000,000	12.0	112,585,054	78.48	784,800	5.5	5.46; 5.48
**Latin American and Caribbean**								
Bahamas	388,000	6000	15.5	224,419	57.84	3470	8.9	8.65; 9.24
Argentina	43,417,000	130,000	3.0	21,313,406	49.09	63,817	1.5	1.46; 1.48
Brazil	207,848,000	800,000	3.8	147,946,206	71.18	569,440	2.7	2.73; 2.75
Chile	17,948,000	57,000	3.2	13,382,824	74.56	42,502	2.4	2.35; 2.39
Ecuador	16,144,000	40,000	2.5	11,659,197	72.22	28,888	1.8	1.77; 1.81
Mexico	127,017,000	210,000	1.7	66,709,328	52.52	110,292	0.9	0.86; 0.87
Panama	3,929,000	24,000	6.1	2,123,625	54.05	12,972	3.3	3.25; 3.36
**Asia and Pacific**								
Kazakhstan	17,625,000	20,000	1.1	14,013,638	79.51	15,902	0.9	0.89; 0.92
Japan	126,573,000	27,000	0.2	65,387,612	51.66	13,948	0.1	0.11; 0.11
Malaysia	30,331,000	81,000	2.7	8,674,699	28.60	23,166	0.8	0.75; 0.77
Nepal	28,514,000	31,000	1.1	19,974,057	70.05	21,716	0.8	0.75; 0.77
Pakistan	188,925,000	120,000	0.6	152,991,465	80.98	97,176	0.5	0.51; 0.52
Singapore	5,604,000	7600	1.4	2,287,553	40.82	3102	0.6	0.53; 0.57
Thailand	67,959,000	520,000	7.7	29,616,532	43.58	226,616	3.3	3.32; 3.35
India ^‡^	1,311,051,000	2,622,102	2.0	831,8618,60	63.45	1,663,724	1.3	1.27; 1.27
Indonesia ^§^	268,000,000	620,000	2.3	59,228,000	22.10 *	137,020	0.5	0.51; 0.51
Vietnam	93,448,000	230,000	2.5	65,712,734	70.32	161,736	1.7	1.72; 1.74
China	1,376,049,000	850,000	0.6	768,452,328	55.84	476,399	0.35	0.35; 0.35
**Oceania**								
New Zealand	4,259,000	3200	0.8	1,085,148	25.48	815	0.2	0.18; 0.2
**All cited countries**	**5,297,987,000**	**18,750,260**	**3.5**	**3,062,858,388**	**57.81**	**5,206,859**	**1.7 ^#^**	**1.7; 1.7**

UN—United Nations. Belgium estimated HIV prevalence in 2015: 1.7. Russian Federation estimated HIV prevalence in 2017: 1.2%. ^‡^ India estimated HIV prevalence in 2017: 0.2%. ^§^ Indonesia estimated HIV prevalence in 2016: 0.4% [19,20,21]. * Indonesia estimated *H. pylori* prevalence in 2015: 22.1% [22]. ^#^ When the relative proportion of each country’s population is taken into account in the estimation.

## Data Availability

All the data were included in the manuscript and in supplementary materials.

## Supplementary Materials

The following supporting information can be downloaded at: https://www.mdpi.com/article/10.3390/microorganisms11040887/s1, Figure S1: Global estimated number of *H. pylori*-HIV co-infections by United Nations regions for the year 2015; Table S1: Global estimate of people living with HIV, H. pylori, and H. pylori co-infection based on available data for both infections in UN regions (in bold) and in UN sub-regions. Table S2: Number and incidence rate and number and death rate from gastric cancer at countries levels [29]. Table S3: Incidence and deaths due to the gastric cancer from 1990 to 2017 at regional and global levels [29].

## References

[1] Marshall B.J., Warren J.R. (1984). Unidentified curved bacilli in the stomach of patients with gastritis and peptic ulceration. Lancet.

[2] Crowe S.E. (2019). Helicobacter pylori Infection. N. Engl. J. Med..

[3] Burgard M., Kotilea K., Mekhael J., Miendje-Deyi V.Y., De Prez C., Vanderpas J., Cadranel S., Bontems P. (2019). Evolution of Helicobacter pylori associated with gastroduodenal ulcers or erosions in children over the past 23 years: Decline or steady state?. Helicobacter.

[4] Choi I.J., Kim C.G., Lee J.Y., Kim Y.I., Kook M.C., Park B., Joo J. (2020). Family History of Gastric Cancer and Helicobacter pylori Treatment. N. Engl. J. Med..

[5] Conteduca V., Sansonno D., Lauletta G., Russi S., Ingravallo G., Dammacco F.H. (2013). Pylori infection and gastric cancer: State of the art (review). Int. J. Oncol..

[6] Venneman K., Huybrechts I., Gunter M.J., Vandendaele L., Herrero R., Van Herck K. (2018). The epidemiology of Helicobacter pylori infection in Europe and the impact of lifestyle on its natural evolution toward stomach cancer after infection: A systematic review. Helicobacter.

[7] Lu Y., Xiao F., Wang Y., Wang Z., Liu D., Hong F. (2022). Prevalence of Helicobacter pylori in Non-Cardia Gastric Cancer in China: A Systematic Review and Meta-Analysis. Front. Oncol..

[8] Smith S.I., Ajayi A., Jolaiya T., Onyekwere C., Setshedi M., Schulz C., Otegbayo J.A., Ndip R., Dieye Y., Alboraie M. (2022). Helicobacter pylori Infection in Africa: Update of the Current Situation and Challenges. Dig. Dis..

[9] Hooi J.K.Y., Lai W.Y., Ng W.K., Suen M.M.Y., Underwood F.E., Tanyingoh D., Malfertheiner P., Graham D.Y., Wong V.W.S., Wu J.C.Y. (2017). Global Prevalence of Helicobacter pylori Infection: Systematic Review and Meta-Analysis. Gastroenterology.

[10] UNAIDS HIV_Estimates_from_1990-to-Present. http://www.unaids.org/sites/default/files/media_asset/HIV_estimates_from_1990-to-present.xlsx.

[11] Deyi V.M., Vanderpas J., Bontems P., Van den Borre C., De Koster E., Cadranel S., Burette A. (2011). Marching cohort of Helicobacter pylori infection over two decades (1988–2007): Combined effects of secular trend and population migration. Epidemiol. Infect..

[12] Maartens G., Celum C., Lewin S.R. (2014). HIV infection: Epidemiology, pathogenesis, treatment, and prevention. Lancet.

[13] Lv F.J., Luo X.L., Meng X., Jin R., Ding H.G., Zhang S.T. (2007). A low prevalence of H pylori and endoscopic findings in HIV-positive Chinese patients with gastrointestinal symptoms. World J. Gastroenterol..

[14] Kang J.S., Lee S.H., Lee S., Kim G.H., Park Y.J., Han I.S., Lee J.E., Lee S.O., Moon C. (2019). Role of Upper Gastrointestinal Endoscopy in Patients with Human Immunodeficiency Virus Infection in the Era of Combination Antiretroviral Therapy. Infect. Chemother..

[15] Nkuize M., De Wit S., Muls V., Arvanitakis M., Buset M. (2010). Upper gastrointestinal endoscopic findings in the era of highly active antiretroviral therapy. HIV Med..

[16] Chehter E.Z., Catapani W.R., Margeotto F.B., Germini D., Henriques A.C. (2014). Helicobacter pylori in the Era of Highly Active Antiretroviral Therapy (HAART): A Review. JSM Gastroenterol. Hepatol..

[17] Nevin D.T., Morgan C.J., Graham D.Y., Genta R.M. (2014). Helicobacter pylori gastritis in HIV-infected patients: A review. Helicobacter.

[18] Arya S., Kaji A.H., Boermeester M.A. (2021). PRISMA Reporting Guidelines for Meta-analyses and Systematic Reviews. JAMA Surg..

[19] Sasse A., Deblonde J., Jamine D., Ost C., Van Beckhoven D. Epidémiologie du SIDA et de L’infection à VIH en Belgique. Situation au 31 Décembre 2016. https://www.sciensano.be/sites/default/files/rapport_vih_sida_2016_web.pdf.

[20] Central Intelligence Agency The World Factbook. https://www.cia.gov/library/publications/the-world-factbook/fields/363rank.html.

[21] Be in the KNOW. HIV and AIDS in Asia & the Pacific Regional Overview. Updated 9 January 2020. https://www.beintheknow.org/understanding-hiv-epidemic/data.

[22] Syam A.F., Miftahussurur M., Makmun D., Nusi I.A., Zain L.H., Zulkhairi, Akil F., Uswan W.B., Simanjuntak D., Uchida T. (2015). Risk Factors and Prevalence of Helicobacter pylori in Five Largest Islands of Indonesia: A Preliminary Study. PLoS ONE.

[23] Lee J.E., Lee S.O., Sim Y.K., Lee S., Kim G.H., Kang J.S., Lee S.H. (2022). Seroprevalence of Helicobacter pylori in human immunodeficiency virus-infected patients in a tertiary care hospital in Busan, South Korea. J. Infect. Chemother..

[24] Kafil H.S., Jahromi F.F., Hajikhani B., Shahin N.P., Aghazadeh M. (2011). Screening for the presence of Helicobacter pylori in stool of HIV-positive patients. J. AIDS HIV Res..

[25] Mesfun M.G., Gliga S., Fuchs A., Orth H.M., Schönfeld A., Luedde T., Feldt T. (2022). Prevalence of H. pylori among asymptomatic HIV-positive and negative individuals in Central Ethiopia and efficacy of eradication therapy. IJID Reg..

[26] Vaira D., Miglioli M., Menegatti M., Holton J., Boschini A., Vergura M., Ricci C., Azzarone P., Mulè P., Barbara L. (1995). Helicobacter pylori status, endoscopic findings, and serology in HIV-1-positive patients. Dig. Dis. Sci..

[27] Mc Loughlin R.M., Sebastian S.S., O’Connor H.J., Buckley M., O’Morain C.A. (2003). Review article: Test and treat or test and scope for Helicobacter pylori infection. Any change in gastric cancer prevention?. Aliment. Pharmacol. Ther..

[28] UNAIDS Annex on Methods. Part I Methods for Deriving UNAIDS HIV Estimates. UNAIDS DATA 2019. https://www.unaids.org/sites/default/files/media_asset/2019-UNAIDS-data_en.pdf.

[29] GBD 2017 Stomach Cancer Collaborators (2020). The global, regional, and national burden of stomach cancer in 195 countries, 1990–2017: A systematic analysis for the Global Burden of Disease study 2017. Lancet Gastroenterol. Hepatol..

[30] Graham D.Y., Malaty H.M., Evans D.G., Evans D.J., Klein P.D., Adam E. (1991). Epidemiology of Helicobacter pylori in an asymptomatic population in the United States. Effect of age, race, and socioeconomic status. Gastroenterology.

[31] Mungazi S.G., Chihaka O.B., Muguti G.I. (2018). Prevalence of Helicobacter pylori in asymptomatic patients at surgical outpatient department: Harare hospitals. Ann. Med. Surg..

[32] Bakka A.S., El-Gariani A.B., AbouGhrara F.M., Salih B.A. (2002). Frequency of Helicobacter pylori infection in dyspeptic patients in Libya. Saudi Med. J..

[33] Malfertheiner P., Megraud F., O’Morain C.A., Gisbert J.P., Kuipers E.J., Axon A.T., Bazzoli F., Gasbarrini A., Atherton J., Grahamet D.Y. (2017). Management of Helicobacter pylori infection-the Maastricht V/Florence Consensus Report. Gut.

[34] Nkuize M., Vanderpas J., Buset M., Gomez-Galdon M., Delforge M., Miendje-Deyi V.Y., Muls V., De Wit S. (2021). Primary antibiotic resistance of Helicobacter pylori isolates is twofold more frequent in HIV-positive than HIV-negative individuals: A descriptive observational study. Microbiologyopen.

[35] Cacciarelli A.G., Marano B.J., Gualtieri N.M., Zuretti A.R., Torres R.A., Starpoli A.A., Robilotti J.G. (1996). Lower Helicobacter pylori infection and peptic ulcer disease prevalence in patients with AIDS and suppressed CD4 counts. Am. J. Gastroenterol..

[36] Lionetti P., Amarri S., Silenzi F., Galli L., Cellini M., de Martino M., Vierucci A. (1999). Prevalence of Helicobacter pylori infection detected by serology and 13C-urea breath test in HIV-1 perinatally infected children. J. Pediatr. Gastroenterol. Nutr..

[37] Aceti A., Celestino D., Pennica A., Leri O., Caferro M. (1990). Antibodies to Helicobacter pylori in HIV infection. Lancet.

[38] Greenland S., Rothman K.J., Greenland S. (1998). Meta-analysis. Modern Epidemiology.

[39] Nagata N., Nishijima T., Niikura R., Yokoyama T., Matsushita Y., Watanabe K., Teruya K., Kikuchi Y., Akiyama J., Yanase M. (2018). Increased risk of non-AIDS-defining cancers in Asian HIV-infected patients: A long-term cohort study. BMC Cancer.

[40] de Vries A.C., van Grieken N.C., Looman C.W., Casparie M.K., de Vries E., Meijer G.A., Kuipers E.J. (2008). Gastric cancer risk in patients with premalignant gastric lesions: A nationwide cohort study in the Netherlands. Gastroenterology.

[41] IARC Lyon (1994). Schistosomes, liver flukes and Helicobacter pylori. IARC Monographs on the Evalutaion of Carcinogenic Risks to Humans.

[42] Grulich A.E., van Leeuwen M.T., Falster M.O., Vajdic C.M. (2007). Incidence of cancers in people with HIV/AIDS compared with immunosuppressed transplant recipients: A meta-analysis. Lancet.

[43] Sung H., Ferlay J., Siegel R.L., Laversanne M., Soerjomataram I., Jemal A., Bray F. (2021). Global Cancer Statistics 2020: GLOBOCAN Estimates of Incidence and Mortality Worldwide for 36 Cancers in 185 Countries. CA Cancer J. Clin..

[44] UNAIDS AIDS-Related Deaths. All Ages Regional Datasheet. https://aidsinfo.unaids.org/.

[45] Ford A.C., Yuan Y., Forman D., Hunt R., Moayyedi P. (2020). Helicobacter pylori eradication for the prevention of gastric neoplasia. Cochrane Database Syst. Rev..

[46] Kowada A. (2019). Cost-effectiveness of Helicobacter pylori test and eradication versus upper gastrointestinal series versus endoscopy for gastric cancer mortality and outcomes in high prevalence countries. Scand. J. Gastroenterol..

[47] Pimentel-Nunes P., Libânio D., Marcos-Pinto R., Areia M., Leja M., Esposito G., Garrido M., Kikuste I., Megraud F., Matysiak-Budnik T. (2019). Management of epithelial precancerous conditions and lesions in the stomach (MAPS II): European Society of Gastrointestinal Endoscopy (ESGE), European Helicobacter and Microbiota Study Group (EHMSG), European Society of Pathology (ESP), and Sociedade Portuguesa de Endoscopia Digestiva (SPED) guideline update 2019. Endoscopy.

[48] Huang H.-L., Leung C.Y., Saito E., Katanoda K., Hur C., Kong C.Y., Nomura S., Shibuya K. (2020). Effect and cost-effectiveness of national gastric cancer screening in Japan: A microsimulation modeling study. BMC Med..

[49] Laszkowska M., Oh A., Hur C. (2020). Screening for Upper Gastrointestinal Malignancies in the United States-Which Immigrant Groups Should Be Considered High-Risk?. Gastroenterology.

[50] Karimi P., Islami F., Anandasabapathy S., Freedman N.D., Kamangar F. (2014). Gastric cancer: Descriptive epidemiology, risk factors, screening, and prevention. Cancer Epidemiol. Biomark. Prev..

