# Supplementary material for: Helicobacter pylori and Human Immunodeficiency Virus Co-Infection: Potential Implications for Future Gastric Cancer Risk
Source: Microorganisms. 2023 Mar 29;11(4):887. doi: 10.3390/microorganisms11040887 (PMC10145129; doi:10.3390/microorganisms11040887)
Supplement: Supplementary file 1 [file microorganisms-11-00887-s001.zip › MDPISupplementals table and figure.pdf]

Supplemental Table S1. Global estimate of people living with HIV, *H. pylori*, and *H. pylori* co-infection based on available data for both infections in UN regions (in bold) and in UN sub-regions

	Population	*HIV*		*Helicobacter pylori*		*Helicobacter pylori – HIV co-infection*	
**UN region and sub-region**	**Size**	**Size**	**Prevalence, ‰**	**Size**	**Prevalence, %**	**Size**	**Prevalence, ‰**
**Sub-Saharan Africa**	342,945,000	9,520,000	27.8	278,373,713	81.17	7,527,255	21.9
Africa - West and Central	288,455,000	2,320,000	8.0	236,067,677	81.84	1,937,175	6.7
Africa – South, and East	54,490,000	7,200,000	132.1	42,306,036	77.64	5,590,080	102.6
**North Africa - Middle East**	109,040,000	24,500	0.2	50,423,474	46.24	13,666	0.1
**North America & South, West, North Europe**	639,947,000	1,717,758	2.7	263,657,561	41.20	706,896	1.1
North America	321,774,000	1,100,000	3.4	114,552,012	35.60	391,602	1.2
North Europe †	24,355,000	32,600	1.3	10,637,350	43.68	16,970	0.7
South Europe†	120,510,000	302,400	2.5	70,090,073	58.16	180,420	1.5
West Europe†	173,308,000	282,758	1.6	68,378,126	39.45	117,904	0.7
**Eastern Europe and Central Asia**	191,136,000	1,039,100	5.4	144,314,193	75.50	812,945	4.3
**Latin America and the Caribbean**	416,691,000	1,267,000	3.0	263,359,005	63.20	831,381	2.0
**Asia and Pacific**	3,593,969,000	5,178,702	1.4	2,061,645,294	57.36	2,866,234	0.8
Oceania	4,259,000	3,200	0.8	1,085,148	25.48	815	0.2

† refers to there is a North-South gradient for H. pylori-HIV co-infection prevalence , with Northern inferior to Southern Europe.

Supplement Table S2. Number and incidence rate and number and death rate from gastric cancer at the country's levels

Supplement Table S3. Incidence and deaths due to gastric cancer from 1990 to 2017 at regional and global levels

Global estimated number of *Helicobacter pylori*-HIV co-infections by United Nations regions for the year 2015

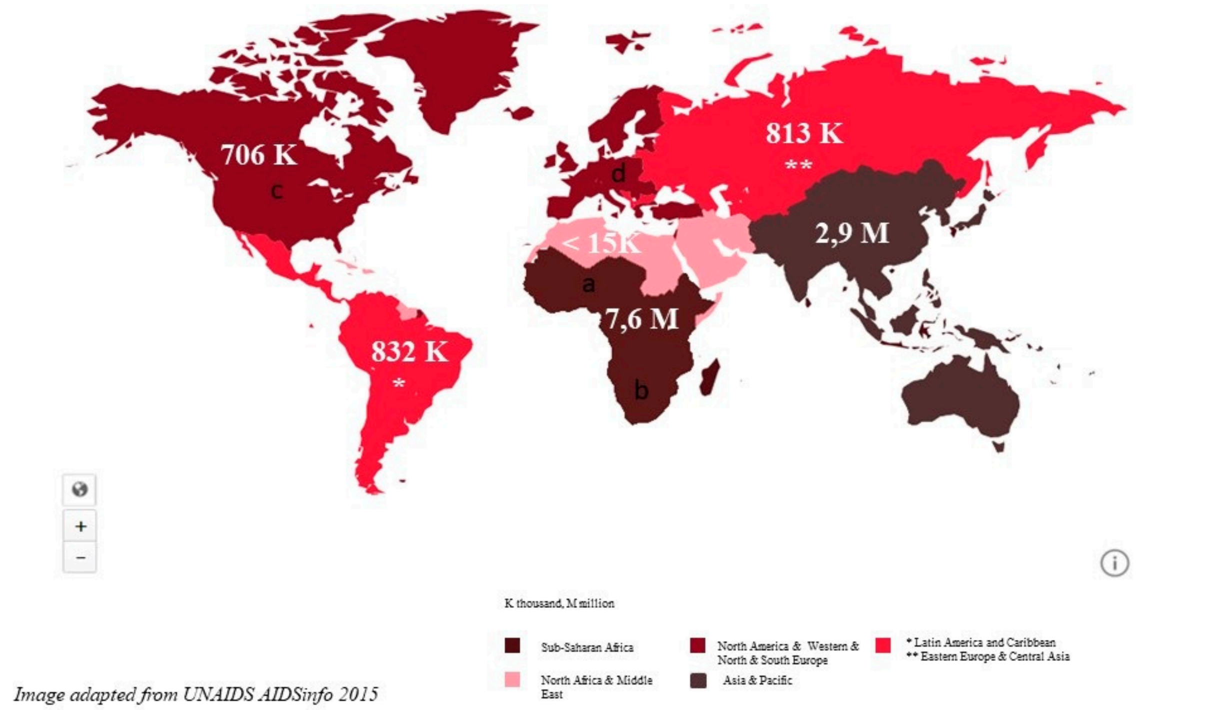

Supplemental Figure S1. This figure summarizes the number of people living with *H. pylori*-HIV co-infection by UN region as follows from top to bottom: sub-Saharan Africa, followed by Asia and Pacific, Latin America and the Caribbeans, Eastern Europe, and North America/North, West, South Europe, and finally North Africa and the Middle East. a central and West Africa, 1.9M, b South Africa, 5.6M, c North America, 316K, d North, South, and West Europe, 315K.
